# *BigBovid*- Evaluation of a Newly Developed 9 mm Bullet-Shooting Stunner for Adequate Stunning of Heavy Cattle

**DOI:** 10.3389/fvets.2022.949198

**Published:** 2022-07-27

**Authors:** Dominic Gascho, Roger Stephan, Clemens Bauer, Michelle Aimée Oesch, Henning Richter

**Affiliations:** ^1^Department of Forensic Medicine and Imaging, Institute of Forensic Medicine, University of Zurich, Zurich, Switzerland; ^2^Institute for Food Safety and Hygiene, Vetsuisse Faculty, University of Zurich, Zurich, Switzerland; ^3^Veterinary Services, Environmental and Health Protection, Zurich, Switzerland; ^4^Scientific Communication and Public Relations, Vetsuisse Faculty, University of Zurich, Zurich, Switzerland; ^5^Diagnostic Imaging Research Unit (DIRU), Clinic for Diagnostic Imaging, Vetsuisse Faculty, University of Zurich, Zurich, Switzerland

**Keywords:** heavy bulls, desensitization, concussion, animal welfare, slaughtering

## Abstract

The stunning of heavy cattle and water buffalo is an animal welfare problem, as conventional cartridge fired captive-bolt stunners are not suitable due to the thicker skull bones and the greater depth of penetration required to reach and damage the relevant brain regions for deep unconsciousness. This current animal welfare problem requires a suitable and feasible as well as commercially available and legally approved stunning device to ensure deep unconsciousness of these animals. In this study, the use of a newly developed bullet-shooting stunner, the *BigBovid*, with two different types of hunting ammunition, namely .3*8 SPL FMJ-TC* and .3*57 MAG FTX*^®^ bullets, was evaluated on 22 heavy cattle (mean weight: 1062.27 kg, standard deviation: 124.09 kg). In ballistic experiments, the *BigBovid* reached a mean energy density of 8.18 J/mm^2^ (mean error: 0.45 J/mm^2^) for the .3*8 SPL FMJ-TC* and 17.56 J/mm^2^ (mean error: 2.67 J/mm^2^) for the .3*57 MAG FTX*^®^. In *in vivo* experiments, the use of the .3*8 SPL FMJ-TC* resulted in overpenetration three times. The .3*57 MAG FTX*^®^ bullets showed to be more advantageous, because on the one hand no overpenetration occurred and on the other hand the bullets fragmented into small parts after penetration into the skull. The fragments were scattered in the brain tissue, such as the thalamus and the brain stem, and thus there is a high probability to damage the brain regions relevant for deep unconsciousness. Based on the results of this study, the use of the *BigBovid* in combination with the .3*57 MAG FTX*^®^ bullet is found to be suitable for stunning heavy cattle.

## Graphical Abstract

The *BigBovid* in combination with *.357 MAG FTX*^®^ *and .38 SPL FMJ-TC* bullets has been tested for stunning heavy cattle. 
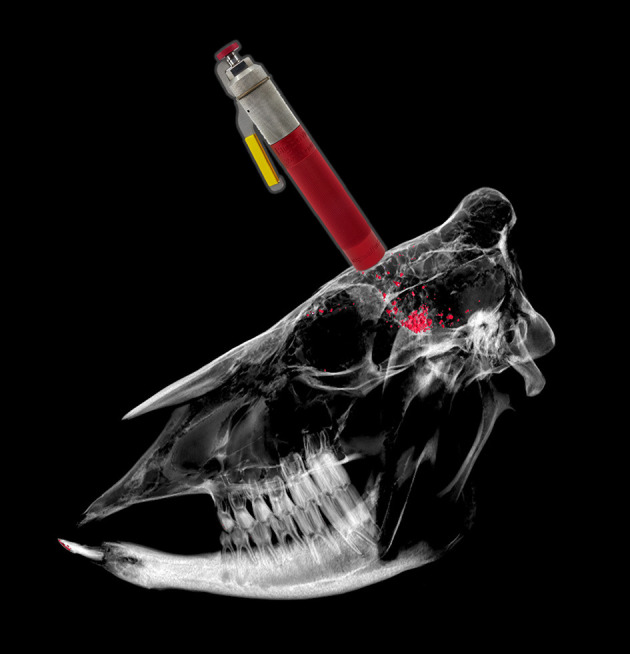

## Introduction

The *European* (1) and *Swiss* (2) legal requirements state that for slaughtering of animals, stunning must lead to deep unconsciousness. The term “deep unconsciousness” refers to the complete loss of the ability to process external stimuli. The animal is therefore no longer sensitive to pain, which is why the term “desensitization” is used for the intended effect of stunning. Desensitization methods relevant to cattle fall into two categories: electrical and mechanical. Electrical methods include head-only stunning and head-to-body stunning. The principle of head-only electrical stunning is the application of a current through the brain sufficient to induce generalized epileptiform activity in the brain so that the animal immediately becomes unconscious, whereas head-to-body electrical stunning refers to the passing of an electrical current through the body to produce cardiac fibrillation (3). This desensitization method will not be discussed further in this study. Mechanical methods addressed in this study include bolt stunning and free projectile stunning using firearms, which are aimed at damaging the brainstem and thalamic region for the purpose of desensitization. The thalamus, along with the cerebral cortex, which form the thalamo-cortical complex, are the important brain regions for consciousness. The functions of the thalamus include the transmission of sensory signals to the cerebral cortex and the regulation of consciousness. The thalamo-cortical complex is regulated by the brainstem, which also controls cardiac and respiratory functions. Therefore, a functioning brainstem and thalamus are essential for rudimentary forms of consciousness (4). Accordingly, damage of the brainstem and thalamic region is the goal to induce rapid loss of consciousness for the stunning of the animals.

In order to damage the brainstem and thalamic region for the purpose of desensitization, dedicated captive-bolt stunners were developed for use on cattle. However, due to their limited penetration capacity and bolt lengths of <120 mm, these common captive-bolt stunners are not suitable for all cattle species. Older heavy bulls differ from common domestic cattle by the larger skull size and increased frontal bone thickness, and in water buffaloes the frontal and paranasal sinuses are generally deeper than in bovines (5). Accordingly, a bolt length of 155 mm is considered necessary to reliably reach the regions of the thalamus in heavy cattle and a bolt length of 172 mm in water buffalo, corresponding to the maximum distances between the frontal distance from the outer table of the compact bone to the thalamus measured in a previous study (5). Therefore, conventional bolt stunning proves unsuitable for stunning heavy cattle and water buffaloes. However, adequate stunning is an essential aspect of animal welfare and legislation. The consequences of an inadequate stunning or slaughter method result in pain and distress for the animal and thus raise ethical questions and further may affect the quality of meat (6–8). This is why an ordinary revolver is often used in *Switzerland* instead of a common captive-bolt stunner (5). However, the use of such an ordinary revolver for stunning is not a method endorsed by the *Federal Food Safety and Veterinary Office*. For this reason, a special bullet-shooting stunner has recently been developed and used to stun water buffaloes, which have thicker frontal bones and larger frontal and nasal sinuses than domestic cattle (9). However, this custom-made 9 mm bullet-shooting stunner never went into production and is therefore not generally available. Ballistic studies of former bullet-shooting stunners, such as the so-called *Humane Killer* which shoots 7.5 mm bullets, showed that this device does not provide the desired penetration capacity, which in this case is not due to the caliber or weight of the bullet but to its low velocity (10). Attempts to develop a bolt gun with a more powerful propelling charge also failed (9). Consequently, there is still no suitable cartridge fired stunner available for adequate stunning of heavy cattle and water buffaloes.

With regard to the evaluation of adequate ammunition for bullet-shooting stunners, ballistic examinations showed that 9 mm semi-jacketed soft-point bullets are preferable to 9 mm full metal jacket bullets, as the latter tend to overpenetrate (10). Pneumatically operated penetration stunning devices with a bolt length of 210 mm at a pressure of 190 psi are available (11). However, these devices are much too expensive for small slaughterhouses. There is a need to develop alternatives for heavy cattle that are more affordable than the powerful pneumatic stunning equipment used on very large abattoirs. Therefore, a bullet-shooting stunner is desired that generates sufficient penetration capacity for a 9 mm bullet to reach the targeted brain region but with a low risk of overpenetration.

In this study, we are evaluating a newly developed bullet-shooting stunner to be used in combination with two types of 9 mm bullets for stunning heavy cattle with thick frontal bones and oversized heads. The objectives are firstly to find out whether this bullet-shooting stunner in combination with the respective ammunition provides the required penetration capacity for the frontal bone thickness and the required penetration depth into the brain tissue up to the thalamus, and secondly, which type of ammunition is more suitable for desensitization in terms of tissue destruction in the thalamus and brainstem and safety in terms of the projectile not exiting the cranial cavity.

## Materials and Methods

### Stunning Gun and Ammunition

**Figure 1:**
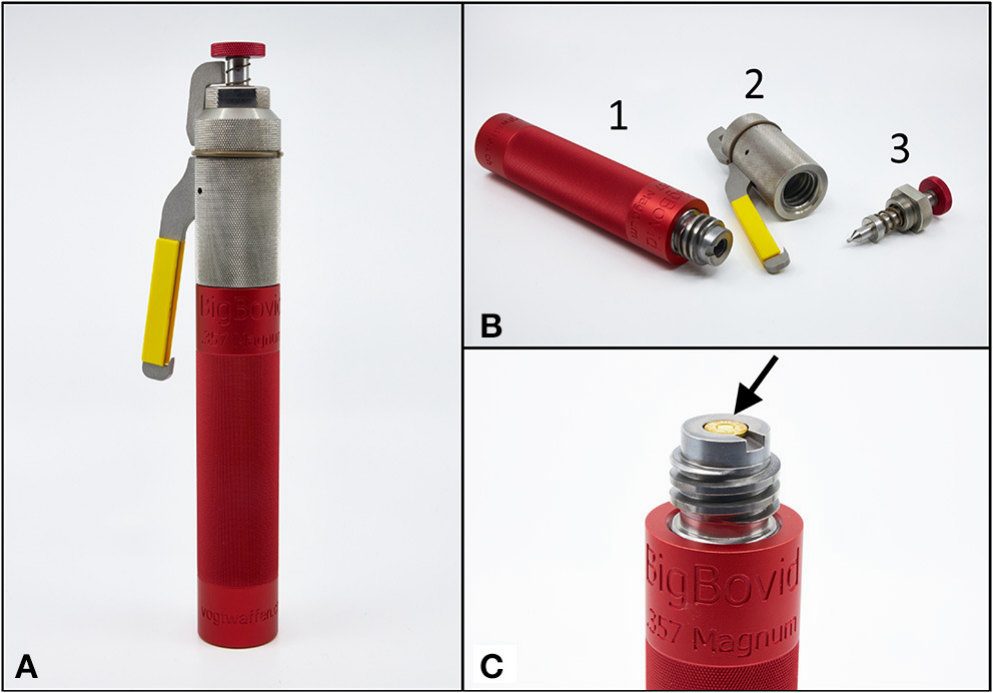
**(A)** In shape and size (total length: 280 mm) the *BigBovid* looks like an ordinary captive-bolt stunner. **(B)** The device consists of three parts, namely the barrel (1), the trigger (2), and the firing pin (3). **(C)** To insert the cartridge into the chamber (arrow), the upper silver end is unscrewed from the lower red end. The two parts are then screwed together again for use on heavy cattle.

The bullet-shooting stunner was explicitly developed for the stunning of heavy cattle and thus bears the designation *BigBovid* (*Vogt Waffen AG, Oberglatt, Switzerland*). The external design of the *BigBovid* corresponds to the shape and size of a typical captive-bolt stunner. The total length of the device is 280 mm, and the total weight is 1700 g. The barrel length is 195 mm. To insert a 9 mm cartridge, the *BigBovid* is screwed on (Figure 1).

**Figure 2:**
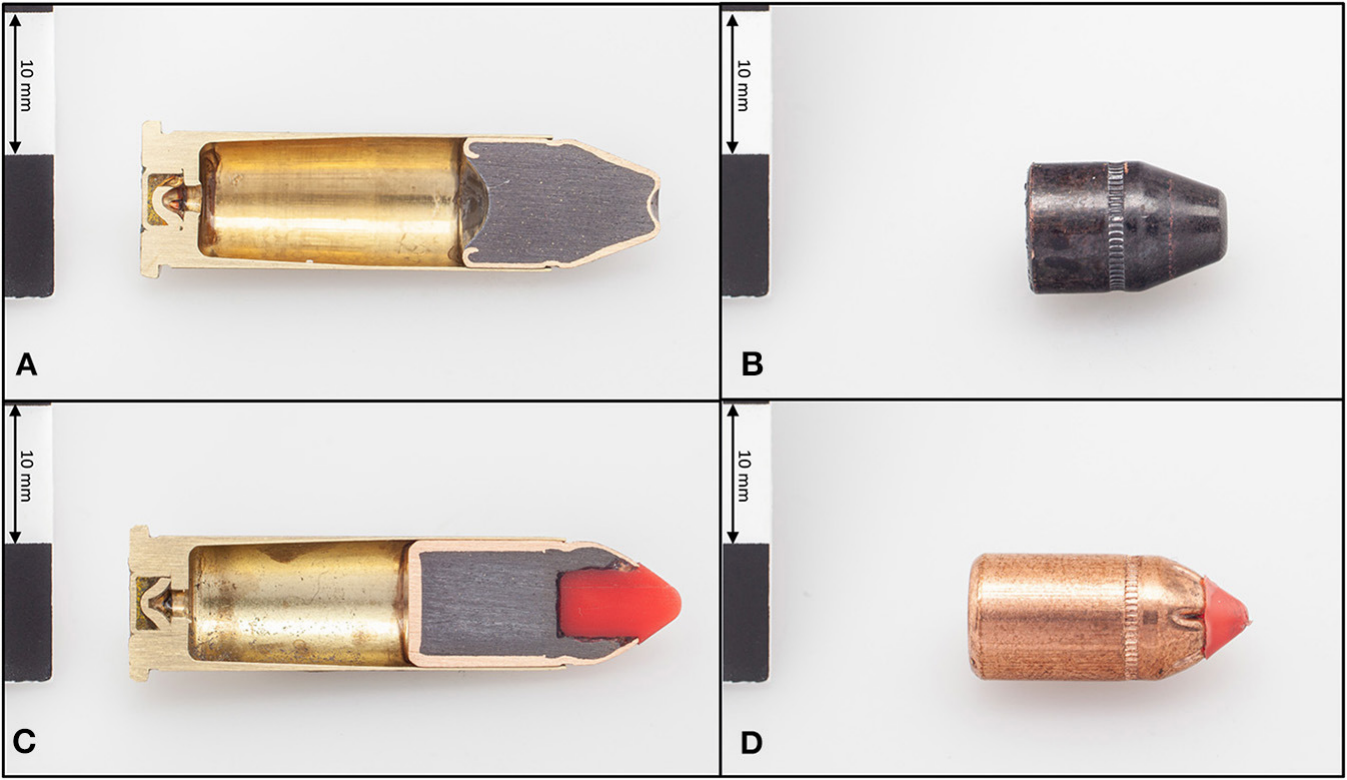
Specimens of a full metal jacket *.38 SPL FMJ-TC* cartridge **(A)** and projectile **(B)** with a truncated cone and a *.357 MAG FTX*^®^ cartridge **(C)** and projectile **(D)** with the special tip, with the cartridges cut open in the center to illustrate the interior.

Based on the recommendations of ballisticians and as a result of test firings on severed cattle heads of slaughtered animals, two different ammunitions were selected for evaluation of the shooting devices (Figure 2). On the one hand, *.38 SPL FMJ-TC* bullets (*Black Mamba, Fiocchi, Lecco, Italy*) with a mass of 110 gr were used. These full-metal-jacket (*FMJ*) bullets with truncated cones (*TC*) are designed for good stopping power. On the other hand, *.357 MAG FTX*^®^ bullets (*Hornady*^®^ *LEVERevolution*^®^*, Grand Island, Nebraska, U.S.A*.) with a mass of 140 gr were used. These bullets have a special tip (*Flex Tip*^®^ Technology) and thus deliver up to 40% more energy than conventional flat-tip bullets. The sectional density, which is the ratio of the mass to the caliber squared, was 0.177 lb_m_/in^2^ for the *.38 SPL FMJ-TC* and 0.157 lb_m_/in^2^ for the *.357 MAG FTX*^®^.

The lower limit was set that the *BigBovid* must achieve at least the same penetrating capacity and a comparable effect as an ordinary revolver using these ammunitions. Therefore, ballistic and *in vivo* experiments were also performed with a conventional 9 mm *COLT King Cobra* revolver using each of the two munitions.

### Ballistic Experiments

Velocities were measured at a short distance using a ballistic velocity measurement system (model: BMC 21a, *Kurzzeit Messtechnik, Schillingsfürst, Switzerland*). For each of the two ammunitions, three velocity measurements were taken with the *BigBovid* and with the revolver respectively (velocity experiments: *n* = 12). The energy density (*E*′), which is the ratio of the kinetic energy (*E*_*kin*_) to the cross-sectional area (*A*), was calculated based on the measured velocity (*v*), the mass (*m*), and caliber (*cal*) of the respective ammunition:


(1) $$\begin{array}{l} E^{{}^{\prime }\;}=\frac{E_{kin}}{A}=\frac{\;\frac{1}{2}m{\;v}^{2}}{{\;\frac{1}{4}cal}^{2}\;\pi } \end{array}$$

To account for the propagation of the uncertainty in the mean velocity ($\bar{v}$) for the calculation of the mean kinetic energy (${\bar{E}}_{kin}$) and finally for the mean energy density (${\bar{E}}^{{}^{\prime }\;}$), the corresponding mean errors were calculated:


(2) $$\begin{array}{lll} \Delta \bar{v} & = & \frac{v_{\max}-v_{\min}}{n} \end{array}$$

(3) $$\begin{array}{lll} \Delta {\bar{E}}_{kin} & = & \frac{2\Delta \bar{v}}{\bar{v}}{\bar{E}}_{kin} \end{array}$$

(4) $$\begin{array}{lll} \Delta \bar{E^{{}^{\prime }}} & = & \frac{1}{A}{\bar{E}}_{kin} \end{array}$$

Instead of additional testing with artificial skin and bone in front of the soap blocks, test trials were conducted on severed cattle heads to ensure the penetrating capacity and penetration depth of the bullets prior to use on live animals. The test trials were conducted on an outdoor shooting range with the heads positioned and secured on a bullet trap mat on the ground. These test series were a condition for the approval of the *Federal Food Safety and Veterinary Office* for the use on live animals during the regular slaughter process.

The measured and calculated values are given as mean value and standard deviation (SD) or mean error. All values are rounded to two decimal places.

### *In vivo* Experiments

A total of 28 heavy cattle were included in this study. The designation “heavy” refers to cattle weighing more than 800 kg for which the legal regulations require the use of bolts of at least 120 mm instead of 80 mm and for which propelling charges or operating pressures with proven sufficient effectiveness must be used in accordance with the manufacturer's specifications (2). The *BigBovid* and the revolver were used for stunning in regular slaughters of heavy cattle performed by the same butcher. After slaughter, the head of each cattle was severed and examined by computed tomography (CT). All CT scans were performed with a standard clinical CT scanner (*SOMATOM*^®^ *Definition Flash, Siemens Healthineers, Erlangen, Germany*). The scan parameters were 120 kVp, 600 mAs, and a pitch of 0.35. Reconstructions were performed with field-of-views (FOV) adjusted to the individual size of the head and of the brain region with a slice thickness of 0.6 mm using a smooth kernel for the assessment of the soft tissue and a hard kernel to enhance edges for the assessment of bones. In addition, extended CT scale reconstructions were computed to assess bullets and bullet fragments (12). Multi-planar reformations were used to align the cattle head data for the corresponding digital measurements of frontal bone thickness, meaning from the outer table to the inner table of compact bone, and penetration depth in sagittal view aligned to the entry hole and the final position of the bullet or bullet fragment. Maximum intensity projections were used to demonstrate the distribution of bullet fragments. Volume rendering technique was used to roughly classify the number of fragments into many, few, and none.

On the CT data, the thickness of the frontal sinuses and the maximum distance of bone fragments perpendicular to the bullet path were measured. Furthermore, CT data were examined for mechanically induced damage to the thalamus and brainstem caused either directly by the bullet or indirectly by the hydraulic pressure generated when the bullet penetrated the tissue. For this purpose, attention was paid to discernible destruction of the tissue by the bullet path and temporary wound cavity, as well as the distribution of bullet and bone fragments. The destruction in the target region, which in this study is the thalmus and brainstem, was graded as follows: 0 = no evidence of destruction, 1 = only gas inclusions indicating tissue laceration and/or possible hemorrhage, 2 = few bullet and/or bone fragments in the target region in addition to grading 1, 3 = multiple bullet and/or bone fragments in addition to grading 1.

In statistical analysis, a *p*-value of <0.05 was considered significant. The Wilcoxon rank sum test was applied to test the heavy cattle in the two *BigBovid* groups (group 1: *.38 SPL FMJ-TC*, group 2: *.357 MAG FTX*^®^) for significant differences in frontal sinus thickness and weight. This test was also used to test whether there were significant differences between the two groups in terms of the maximum distance between bone fragments and in terms of the graduation of destruction in the target region. Statistical analysis was performed using the computing environment *R* (*R Development Core Team Vienna, Austria*).

## Results

The results of the ballistic examinations are summarized in Table 1.

**Table 1:** Ballistic data, given as mean value and mean error.

**Device**	**Ammunition**	**$\bar{v}\left(\Delta \bar{v}\right)$[m/s]**	**${\bar{E}}_{kin}\left(\Delta {\bar{E}}_{kin}\right)$[J]**	**${\bar{E}}^{\prime }\left(\Delta {\bar{E}}^{\prime }\right)$[J/mm^**2**^]**
*BigBovid*	*.38 SPL FMJ-TC*	384.79 (1.48)	527.69 (4.05)	8.18 (0.45)
*COLT King Cobra*	*.38 SPL FMJ-TC*	235.67 (12.15)	198.73 (20.49)	3.08 (2.26)
*BigBovid*	*.357 MAG FTX^®^*	499.89 (5.33)	1133.63 (24.19)	17.56 (2.67)
*COLT King Cobra*	*.357 MAG FTX^®^*	425.65 (4.45)	821.88 (17.18)	12.73 (1.89)

*$\bar{v}$, mean velocity; $\Delta \bar{v}$, mean error of the velocity measurements; ${\bar{E}}_{kin}$, mean kinetic energy; $\Delta {\bar{E}}_{kin}$, mean error of the kinetic energy calculations; $\bar{E^{\prime }}$, mean energy density; $\Delta \bar{E^{\prime }}$= mean error of the energy density calculations*.

The *BigBovid* produces a higher bullet velocity compared to the revolver, resulting in a higher kinetic energy and energy density for the respective bullets shot with the *BigBovid*. For the *.38 SPL FMJ-TC* the mean energy density is about 2.5 times higher and for the *.357 MAG FTX*^®^ about 1.5 times higher when fired with the *BigBovid* compared to the revolver. With regard to the use of the *BigBovid*, the mean velocity of the heavier *.357 MAG FTX*^®^ was higher than that of the lighter *.38 SPL FMJ-TC*, resulting in a mean energy density for the *.357 MAG FTX*^®^ that is about two times higher than that of the *.38 SPL FMJ-TC*.

In the test series on severed cattle heads, both types of ammunition perforated the frontal bones in three experiments each, and in a subsequent CT the bullet fragments showed up in the posterior cranial fossa or the bullet was not detected because it had exited the back of the skull. These qualitative results were considered satisfactory in terms of penetration capacity and penetration depth, and the use of the *.38 SPL FMJ-TC* and the *.357 MAG FTX*^®^ in combination with the *BigBovid* and the *COLT King Cobra* revolver for desensitization of live animals in the context of regular slaughter was approved by the responsible authority.

Using the *BigBovid* on 22 heavy cattle with a mean weight of 1062.27 kg (SD: 124.09 kg), a total of 10 stuns were performed with the *.38 SPL FMJ-TC* and 12 stuns with the *.357 MAG FTX*^®^. There is no significant difference between the two groups in terms of weight (*p* > 0.8). With mean values of 2.60 cm (SD: 0.61 cm) and 2.52 cm (SD: 0.37 cm) the thickness of the frontal bone was also similar between the groups (*p* > 0.4). In all stunned cattle, adequate stunning was confirmed. On the one hand, the regulations for complete unconsciousness were fulfilled according to the *European* (1) and *Swiss* (2) legal requirements and taking into account the guidelines of the *European Food Safety Authority* (13) and the *American Veterinary Medical Association* (14). This is defined by immediate collapse, tonic spasms followed by a clonic phase, absence of respiration, absence of corneal reflex, no rotation of the eyeball, no response to pain stimulus, no vocalizations, no directional movements or attempts to stand up. On the other hand, postmortem computed tomography was performed in all stunned cattle, which revealed injuries in the thalamic region, confirming adequate stunning. The graduation of destruction in the thalamus and brainstem was significantly higher when the *BigBovid* was used with the *.357 MAG FTX*^®^ than when it was used with the *.38 SPL FMJ-TC* (*p* < 0.031). Also the maximum distance of the bone fragments was significantly higher in the the *.357 MAG FTX*^®^ group than in the *.38 SPL FMJ-TC* group (*p* < 0.01). Graduations and measurements are summarized in Table 2.

**Table 2:** *BigBovid* data.

**No**.	**Breed**	**Sex**	**Age** **[years] +[months]**	**Weight** **[kg]**	**Ammunition** **(group)**	**Bone** **thickness** **[cm]**	**Penetration** **depth** **[cm]**	**Maximum** **distance of** **bone fragments** **[cm]**	**Destruction in** **the target** **region**
1	*Limousin*	male	6 + 9	1,200	*.38 SPL FMJ-TC*	2.65	21.25^*^	2.54	1
2	*Salers*	male	5 + 5	1,177	*.38 SPL FMJ-TC*	4.14	o.p.	2.31	2
3	*Angus*	male	3 + 11	931	*.38 SPL FMJ-TC*	2.61	13.29	5.06	2
4	*Simmentaler*	male	4 + 3	1,180	*.38 SPL FMJ-TC*	2.66	18.73^*^	2.44	1
5	*Angus*	male	3 + 3	970	*.38 SPL FMJ-TC*	2.60	18.24^*^	1.39	1
6	*Angus*	male	4 + 6	990	*.38 SPL FMJ-TC*	2.67	9.36	3.01	3
7	*Limousin*	male	4 + 8	1,121	*.38 SPL FMJ-TC*	2.05	o.p.	3.83	2
8	*Angus*	male	6 + 4	918	*.38 SPL FMJ-TC*	2.34	11.52	4.68	3
9	*Limousin*	male	4 + 4	1,110	*.38 SPL FMJ-TC*	1.59	o.p.	2.34	2
10	*Simmentaler*	male	2 + 10	900	*.38 SPL FMJ-TC*	2.67	13.62	1.18	2
11	*Charolais*	male	4 + 0	1,302	*.357 MAG FTX^®^*	2.65	12.2	7.08	2
12	*Simmentaler*	male	4 + 2	1,110	*.357 MAG FTX^®^*	2.48	9.84	4.61	2
13	*Limousin*	male	4 + 0	1,067	*.357 MAG FTX^®^*	2.51	14.38	7.25	3
14	*Angus*	male	4 + 0	1,138	*.357 MAG FTX^®^*	2.98	9.37	5.82	2
15	*Limousin*	male	4 + 6	1,300	*.357 MAG FTX^®^*	2.42	14.28	6.07	3
16	*Simmentaler*	male	3 + 8	1,095	*.357 MAG FTX^®^*	2.51	11.5	5.94	3
17	*Limousin*	male	5 + 7	1,045	*.357 MAG FTX^®^*	2.15	13.03	7.89	3
18	*Hereford*	male	3 + 11	1,013	*.357 MAG FTX^®^*	2.42	13.66	6.86	3
19	*Limousin*	male	5 + 1	1,083	*.357 MAG FTX^®^*	2.09	11.84	6.72	2
20	*Limousin*	male	3 + 6	933	*.357 MAG FTX^®^*	1.96	14.07	8.23	3
21	*Hereford*	male	5 +11	958	*.357 MAG FTX^®^*	3.37	13.06	6.55	3
22	*Angus*	male	3 + 8	828	*.357 MAG FTX^®^*	2.64	12.01	6.79	2

*o.p., overpenetration*;

* *, perforation of the posterior cranial fossa*.

**Figure 3:**
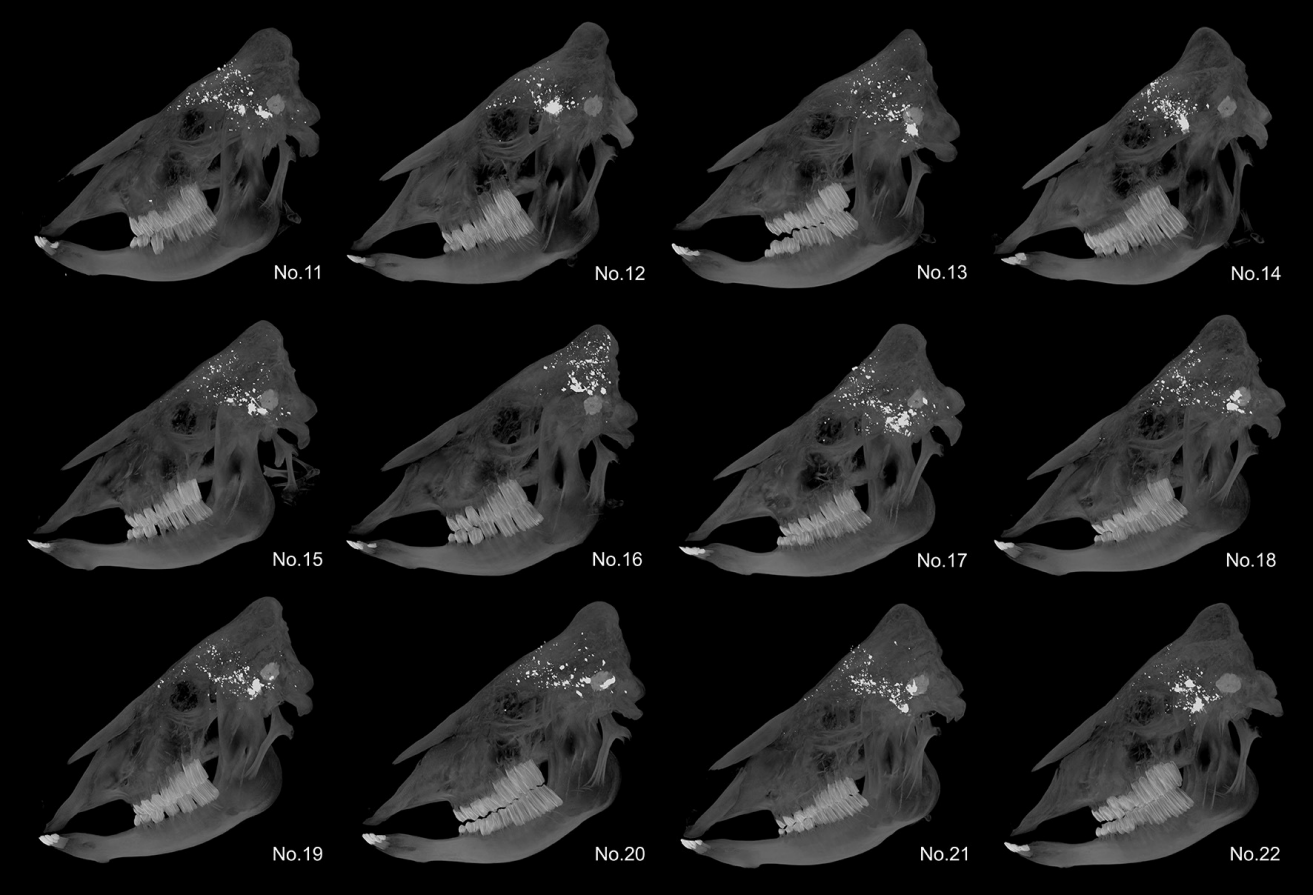
Maximum intensity projection of heavy cattle skulls in the *.357 MAG FTX*^®^ group in sagittal view to demonstrate bullet fragmentation and distribution of fragments inside the cranial cavity.

For the *.357 MAG FTX*^®^, all bullets were fragmented and the most deeply penetrated fragments were in the posterior cranial fossa in each case. The mean value of the penetration depth was 12.44 cm (SD: 1.57 cm). Inside the cranial cavity the fragments were widely distributed (Figure 3).

In the case of the *.38 SPL FMJ-TC*, four bullets were also in the posterior cranial fossa. However, six bullets penetrated the posterior cranial fossa, of which three were still in the soft tissues of the head but the remaining three were not detected on CT and therefore identified as overpenetrated. Less meaningful, therefore, is the mean value of the penetration depth of the lodged bullets, which was 11.95 cm (SD: 16.69 cm) and 19.41 cm (SD: 1.32 cm), respectively, for the bullets that had lodged in or perforated the posterior fossa but then lodged in soft tissue.

**Figure 4:**
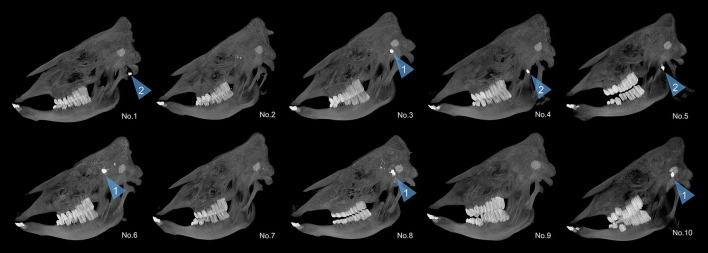
Maximum intensity projection of heavy cattle skulls in sagittal view for demonstration of bullet end positions. In No. 3, 6, 8, and 10, the *.38 SPL FMJ-TC* was lodged in the posterior cranial fossa of the skull (arrowhead 1). In No. 1, 4, and 5, the bullet perforated the posterior cranial fossa but eventually remained lodged in the soft tissue of the skull (arrowhead 2). In Nos. 2, 7, and 9, the bullet did not show up on CT scans and overpenetration was defined. It should be noted that the distribution of the bone fragments inside the cranial cavity is not visible on these reconstructions.

Using the *COLT King Cobra* revolver on six heavy cattle with a mean weight of 959.92 kg (SD: 134.39 kg), a total of three stuns were performed with the *.38 SPL FMJ-TC* and also three stuns with the *.357 MAG FTX*^®^. Conducting three stun shots for each ammunition was considered sufficient for comparison, since the ballistic characteristics as well as the practical use of the revolver are already known. The heavy cattle had a mean frontal bone thickness of 2.03 cm (SD: 0.29 cm) when stunned with the *.38 SPL FMJ-TC* and of 2.40 cm (SD: 0.65 cm) when stunned with the *.357 MAG FTX*^®^. The *.38 SPL FMJ-TC* reached a mean penetration depth of 10.57 cm (SD: 2.08 cm) and the *.357 MAG FTX*^®^ a mean penetration depth of 11.72 cm (SD: 1.57 cm). Thus, the *BigBovid* achieved a mean penetration depth that was just 1.11 and 1.06 times greater for the *.38 SPL FMJ-TC* and *.357 MAG FTX*^®^ bullets, respectively, than that achieved with the revolver and the corresponding ammunition. With regard to the *.38 SPL FMJ-TC*, no or only a few small fragments were detected in each cattle head (Figure 3). In contrast, the *.357 MAG FTX*^®^ fragmented each time into many small fragments (Figure 4).

## Discussion

The results of this study show that the *BigBovid* generates sufficient penetration capacity for the selected *.38 SPL FMJ-TC* and *.357 MAG FTX*^®^ to penetrate the frontal bone structures of heavy cattle and cause damage to the brain structures commensurate with adequate stunning. Of the ammunition chosen, the *.357 MAG FTX*^®^ is preferable to the *.38 SPL FMJ-TC* because of more massive destruction of brain tissue. In view of these results, the *BigBovid* is recommended as a suitable tool for the desensitization of heavy cattle because the *.357 MAG FTX*^®^ causes significantly greater destruction of the thalamus and brain region.

The destruction of the brain regions responsible for the process of the external stimuli, such as the thalamus and brainstem, is essential for the welfare of the animal at slaughter (8). For this purpose, the *BigBovid* was designed to be similar in shape and size to an ordinary captive-bolt stunner, so that the correct position and orientation of the device for adequate stunning can be achieved by the butcher in the usual way. Like an ordinary captive-bolt stunner, the butcher can press the *BigBovid* directly against the forehead of the cattle and thus, with practiced handling, perform the stunning. Or a shot can be fired from a short distance as this has been done so far when using the revolver. However, using the revolver, the angle of impact must be adjusted with the wrist and the body position for such an aimed shot additionally complicates the firing of the shot at the intended angle of impact. Of course, there are also captive-bolt stunners that are similar in shape to a handgun, but these are pressed directly against the head, according to the handling of a captive-bolt stunner. For the stunning of heavy cattle, a more or less vertical angle of impact is required, for which the butchers preferred the shape and size of a conventional rod-shaped stunner over a handgun-shaped stunner. The design of the *BigBovid*, which corresponds to that of an ordinary rod-shaped captive-bolt stunner, allows for usually handling when stunning heavy cattle at slaughter. This design was also preferred by the *Federal Food Safety and Veterinary Office*, since the rod-shaped design and lack of a sight make it rather unsuitable for aimed shots from a greater distance as is the case with ordinary handguns. Thus, the *BigBovid* is much reduced to its intended purpose, the stunning of heavy cattle. This certainly also facilitates its approval through the *Federal Food Safety and Veterinary Office* for routine use. Furthermore, the *BigBovid* was provided with a red signal color to prevent confusion with conventional captive-bolt stunners. Nevertheless, for its use a legally required firearms acquisition license is necessary.

A disadvantage of the *BigBovid* compared to a revolver is the fact that it can only be loaded for one shot. However, if an initial shot does not lead to adequate stunning, a quick second shot is necessary. Therefore, it is necessary to have a second *BigBovid* ready when a second shot is required, as is the case when using conventional captive-bolt stunners. However, due to the penetration depth of the bullets and their destructive potential when using the *BigBovid*, a second shot is expected to be less frequent than with conventional cartridge fired captive-bolt stunners, where a second shot is frequently necessary (15).

With regard to the ammunition, the overpenetration of the *.38 SPL FMJ-TC* in three out of 10 cases is a clear disadvantage compared to the *.357 MAG FTX*^®^ where no overpenetration was observed. When the bullet exits the cattle, overpenetration causes a danger for the employees in the slaughterhouse. For this reason, the use of the *BigBovid* in combination with the *.38 SPL FMJ-TC* was stopped after 10 stuns. Given that overpenetration did not occur using the revolver, and given that the *BigBovid* produced about 2.5 times the energy density for the *.38 SPL FMJ-TC*, a modification to the *BigBovid* would be one way to reduce the likelihood of such overpenetration. For example, the bullet velocity is related to the length of the barrel. The size and design of the *BigBovid* results in a correspondingly long barrel length, which also results in the high velocities of the bullets, which ultimately led to the increased energy density of the bullet when the *BigBovid* was used compared to the revolver. However, a bullet like the *.357 MAG FTX*^®^ is preferable to the *.38 SPL FMJ-TC* anyway. With the *.357 MAG FTX*^®^, which achieved the highest energy density values, no overpenetration was detected due to the fact that the bullets split into many fragments immediately after penetration.

Furthermore, the multiple small fragments of the *.357 MAG FTX*^®^ along with the bone splinters were distributed throughout the brain tissue, making the probability of injuring the thalamus and brainstem by a bullet fragment or bone splinters very high, which is shown by the high graduations of tissue destruction in this study. Thus, the *BigBovid* with the *.357 MAG FTX*^®^ offers a clear advantage over conventional captive-bolt stunners, which are less precise in terms of damaging the corresponding brain regions (15, 16). In ballistic experiments it is shown that the temporary wound cavity caused by a captive-bolt stunner in ordnance gelatin narrows in depth (17), and therefore the bolt must penetrate the brain tissue, if not directly, at least near the relevant brain regions. In terms of penetration depth, the fragments of the *.357 MAG FTX*^®^ had a minimum value of 9.37 cm and a maximum value of 14.38 cm. It should be mentioned here that a bullet does not usually penetrate tissue in a perfectly straight line, so the measured distance from the entry hole to the final position of the bullet or bullet fragment, which is given here as the penetration depth, does not necessarily correspond to the path length of the bullet through the tissue. Regardless, the corresponding fragment was located in the posterior fossa in all of these measurements. This suggests that the bullet fragments would penetrate soft tissue even deeper. This means that the *BigBovid* in combination with the *.357 MAG FTX*^®^ would also be suitable for testing on water buffalo, where a penetration depth of 18 cm must be achieved due to the anatomy of the skull (5).

For the location of a lodged bullet or the visualization of the distribution of bullet fragment, CT is a suitable examination tool (12). This imaging tool also facilitated the examination of the severed heads with regard to cerebral injuries as well as bone thickness and penetration depth measurements. The three-dimensional data can be rotated using multi-planar reformations for any view. The volume rendering technique, in turn, allows the easy detection of radiopaque materials such as, in this case, bullets and bullet fragments.

A limitation of this study is the small number of heavy cattle that were examined in this study. This is due to the expense of a CT scan, which, however, allows impressive results on the three-dimensional distribution of bones and projectile fragments, which was explicitly investigated in this study. Regardless of the small number, there are clear differences between the types of ammunition. Another limitation of this study is that due to the small number of cases, only a single experienced butcher was assigned to use the *BigBovid*. Thus, no statement can be made in this study about the additional factors of experience and skill of the butcher.

Now that the *BigBovid* has been evaluated in an initial round using CT, its use under commercial conditions in large numbers of cattle is required to collect further data on its efficacy.

## Conclusion

The use of the *BigBovid* in combination with the *.357 MAG FTX*^®^ is suitable for stunning heavy cattle. This newly designed bullet-shooting stunner produces a sufficiently high velocity for the bullet to perforate the thick frontal bones of the head of heavy cattle. The *.357 MAG FTX*^®^ bullet fragments immediately, causing the numerous fragments to disperse in the brain tissue, and injury to the thalamic region and the brainstem can be achieved with a high probability. Due to the rapid fragmentation of these bullets when perforating the frontal bone, there is a low risk of overpenetration. The results of this study have been submitted to the Federal Food Safety and Veterinary Office and official approval to use the *BigBovid* has been requested. The legal aspects for approvals in other countries are not the subject of this work.

## Data Availability Statement

The data supporting the conclusions of this article can be requested from the authors.

## Ethics Statement

Ethical review and approval was not required for the animal study because all heavy cattle were desensitized with the *BigBovid* or the revolver during routine slaughter. The examination of the remains of animal carcasses which are not further used for food production, in this case the severed head, is not subject to ethical approval. The study was approved by the Federal Food Safety and Veterinary Office.

## Author Contributions

DG: conceptualization, methodology, investigation, ballistic analysis, writing—original draft, and visualization. RS: conceptualization, writing—review and editing. CB: conceptualization. MO: visualization. HR: conceptualization, methodology, investigation, project administration, writing—review and editing. All authors contributed to the article and approved the submitted version.

## Conflict of Interest

The authors declare that the research was conducted in the absence of any commercial or financial relationships that could be construed as a potential conflict of interest.

## Publisher's Note

All claims expressed in this article are solely those of the authors and do not necessarily represent those of their affiliated organizations, or those of the publisher, the editors and the reviewers. Any product that may be evaluated in this article, or claim that may be made by its manufacturer, is not guaranteed or endorsed by the publisher.
